# Advances in vaccine development through the controlled human infection models for hookworm and schistosomiasis

**DOI:** 10.1371/journal.pntd.0014137

**Published:** 2026-04-02

**Authors:** Marie-Astrid Hoogerwerf, Moses Egesa, Selidgji Todagbe Agnandji, Alex Loukas, Meta Roestenberg

**Affiliations:** 1 Leiden University Center for Infectious Diseases, Leiden University Medical Center, Leiden, The Netherlands; 2 Vaccine Research Theme, Medical Research Council/Uganda Virus Research Institute and London School of Hygiene & Tropical Medicine Uganda Research Unit, Entebbe, Uganda; 3 Department of Infection Biology, London School of Hygiene & Tropical Medicine, London, United Kingdom; 4 Centre de Recherches Médicales de Lambaréné, Lambaréné, Gabon; 5 Institute of Medical Microbiology, University Hospital Münster, Münster, Germany; 6 Australian Institute of Tropical Health and Medicine, James Cook University, Cairns, Australia; The University of Melbourne, AUSTRALIA

## Abstract

Controlled human infection models can play an important role in vaccine development, particularly for neglected tropical diseases such as helminth infections. Currently, controlled infection models have been established for schistosomiasis and hookworm. This review highlights the developments in the controlled human schistosomiasis infection model (CHI-S) and the controlled human hookworm infection model (CHHI) and their contributions to vaccine development. In general, both models are considered safe and well-tolerated. Measures to decrease risk of potential adverse events were taken when developing the models. For both models, production of challenge agents follows the principles of Good Manufacturing Practice. Both models have proven to reliably detect infection and can be used to assess efficacy of immunization strategies. While hookworms and schistosomes are both helminths, the controlled human infection (CHI)-studies have also highlighted differences between these pathogens. Notably, schistosomiasis seems to induce more, dose-dependent, systemic symptoms, whereas in hookworm models skin symptoms are much more prominent. Infection levels for schistosomiasis are therefore limited and lower than those usually seen in endemic populations, whereas for hookworm it is possible to reach levels comparable to mild-moderate intensity infection in the field. Host responses to short-term infection were also different: short-term schistosome infection induced immune-tolerance, whereas short-term infection with hookworm larvae seems to induce a more pro-inflammatory response compared to that seen in the adult worms. Most studies have been performed in naïve non-endemic populations, however, currently the models are being expanded to endemic areas. This has raised new questions around the impact of non-native strains of parasites or vectors to the endemic parasite strains and the environment. Studies in endemic areas, however, will significantly contribute towards understanding the immunology of these helminth infections in pre-exposed individuals. In general, the success of these established models is encouraging to the further development of controlled human helminth infection models.

## Introduction

Controlled human infection models (CHIMs) have been applied since the beginning of the concept of vaccination itself, through the inoculation with cowpox to protect against smallpox by Edward Jenner [1]. Through its history, controlled human infections have greatly contributed to vaccine development for an array of pathogens. For example, the initial proof of concept for the first licensed malaria vaccine RTS,S, was provided through controlled human malaria infection studies [2]. Vaxchora, an oral live cholera vaccine [3] was licensed for travelers based on the proven efficacy in a CHIM.

For vaccine development efforts for neglected tropical diseases (NTDs), where research funding is often limited, the increased cost-effectiveness of CHIMs in the product development pipeline provides an attractive opportunity to vaccine developers. CHIM offer an early proof of concept of efficacy for novel vaccines and drug compounds, consequently derisking overall product development. Due to the controlled and thus synchronized nature of the infection, small groups of participants are sufficient to offer an initial indication of product efficacy and safety without having to set up large-scale phase II/III field studies that rely on natural exposure which can take months or years depending on infection rates and transmission patterns. This results in a significant reduction of clinical trial costs [4].

Helminth infections form a large part of the NTDs which still carry a substantial global burden of morbidity and mortality. Intestinal helminth infections and schistosomiasis together cause a loss of over 3 million disability-adjusted life years (DALY’s), a metric that is forecast to be only partially reduced to 2.5 million in 2050, even with interventions targeting sanitation and hygiene [5]. Although mass drug administration efforts have reduced the burden of disease, reinfection is frequent, and successful elimination has not yet been achieved. Highly potent vaccines are urgently needed to break the cycle of transmission and gain control in many tropical areas in the global south. There are currently no licensed vaccines available against these infections [6,7]. Research into novel helminth vaccines is further hampered by lack of funding, lack of knowledge on how (and if) protective immunity can be elicited and the complex lifecycle of these parasites. Natural infection does not result in protective immunity, possibly due to the immunomodulatory properties of the adult worms [8]. Furthermore, in natural infections people are often co-infected with multiple pathogens and extensively pre-exposed, clouding immune responses and making it virtually impossible to uncover correlates of protection. In a CHIM, studies can be performed in naïve populations, exposed populations without relevant co-infections, and in highly endemic populations. Comparing responses between these different populations allows for a better understanding of tolerance and immunity to helminths. A CHIM study can furthermore provide a direct assessment of infection and of the protective effect of a vaccine compared to large-scale, time-consuming, and expensive field trials [9,10].

There are currently two CHIMs using helminth inocula. For hookworm, a controlled infection model has been applied since the beginning of the 20th century, with initial studies investigating the infective properties of the larvae [10]. The model then received substantial attention as a possible therapeutic strategy against polycythemia vera and later inflammatory disorders such as asthma, celiac disease, and most recently, metabolic syndrome [11–13]. Over the past decade, studies have been undertaken to further refine the hookworm model to test vaccine- and drug efficacy [10,14,15]. The schistosomiasis CHIM has, in contrast, only recently been developed, with the first study performed in 2017 [16].

There are multiple other helminth infections that cause significant morbidity and for which no vaccines are available, such as *Strongyloides*, *Ascaris*, and whipworm. For these, however, there are no CHIM available. This may be due to difficulty culturing the pathogen in a laboratory setting, lack of research funding or concerns about safety and feasibility of the model (e.g., *Strongyloides*). For whipworm (*Trichuris trichiuria*), the first steps towards establishing a controlled human infection model are now being undertaken (NCT05706116).

For both the establishment and refinement of CHIM, there are four key elements to consider. Firstly, a CHIM should be safe to both participants and bystanders. This means that there should be no non-reversible pathology caused by the infection and that either treatment is available, or the condition is self-limiting. Spread of infection must be evaluated and if necessary, precautions must be taken to avoid infection of bystanders. For both safety and reproducibility, challenge material should be produced according to the principles of Good Manufacturing Practice (GMP), although full GMP is often not possible due to the requirements of the culturing process and starting materials [17,18]. Furthermore, the infection needs to be detectable to determine infection rates preferably quantitatively, but also to follow-up clearance of infection after treatment. There must also be a clear scientific rationale for the CHIM study, paying attention to maximizing the utility of any trial by not only exploring the primary endpoint but also considering other possible scientific questions that can be answered from a single CHIM.

In this narrative review, we will discuss the steps taken in the development of the hookworm and schistosomiasis CHIM and how these steps contribute to vaccine development for these pathogens.

## Method

Literature was searched in the PubMed database used the following search terms:

((schistosom*) OR (hookworm OR Necator OR Ancylostoma) OR (helminth*) OR (Trichuris)) AND (“experimental infection*” OR “human challenge” OR “challenge study” OR “challenge model” OR “human infection” OR “infection model” OR “volunteer study” OR “infection in volunteers” OR “volunteer challenge” OR “controlled human infection”). Papers were assessed on relevance to the review, from which a narrative review on model development was compiled. As no paper or protocol has yet been published on the *Trichuris* model, this has not been taken forward into the review. Number of trials, participants, and key findings of the studies are summarized in Table 1.

**Table 1:** Summarizing findings to inform vaccine research.

	Schistosomiasis	Hookworm
No of trials reported	3	18
Estimated number of participants included	44	294^*^
Countries where model is used	The Netherlands, Uganda	Australia, Gabon, New Zealand, The Netherlands, United Kingdom, United States
Key findings from CHIM trials	• Acute schistosomiasis syndrome not related to eggs • Female worms are less susceptible to praziquantel treatment • Repeated exposure does not induce a protective effect	• Exposure to early-stage infection using irradiated larvae or abrogated infection may induce a protective effect • Improvements seen in diseases outcomes for several auto-immune of inflammatory diseases
Key immunologic findings	• Early Th1 response after infection, within 8 weeks after infection followed by Th2 and Treg response. • Increase in IgG1, not inducing protection in repeated infection study.	• Association of IgG1 with decreased egg load. • Mixed Th1 and Th2 cytokine responses after larval exposure and challenge
Anticipated number of participants in vaccine trial	• Proposed vaccine-CHI trial: 24 participants per group to detect 50% reduction in CAA positivity (NCT 05999825)	• Assuming 50% reduction in egg load, using 5 repeated samples, groups of 6 volunteers can reach 80% power.
Vaccines tested using CHIM	• Protocol under preparation for first vaccine-CHIM trial using Sm-p80 vaccine (NCT 05999825)	• 1 trial ongoing using GST-1-vaccine (NCT03172975)

* Since 1984. Estimated number of subjects including historical experiments starting from 1901 ± 500 (Chapman and colleagues PlosNTD 2021).

## The controlled human schistosomiasis infection model

**Fig 1:**
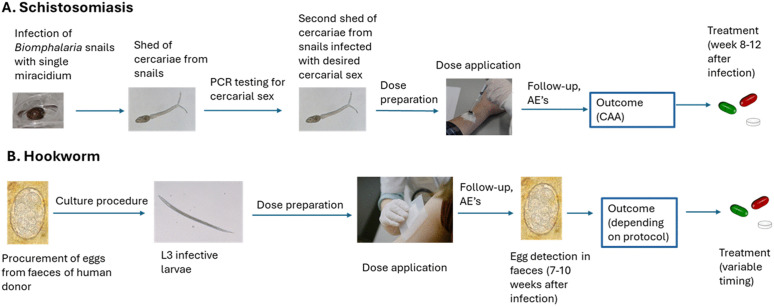
Flow of the controlled schistosoma model (A) and the hookworm model (B). Clipart made using openclipart.org. Photographs taken at Leiden University Center for Infectious Diseases, Leiden University Medical Center, the Netherlands.

Schistosomiasis affects over 240 million people worldwide [19]. Its main pathology is caused by the deposition of eggs, either in the bladder or in the portal vasculature of the liver, depending on the *Schistosoma* species. This leads to a chronic inflammatory process, ultimately resulting in liver cirrhosis, bladder carcinoma and in female or male genital schistosomiasis, the first of which is associated with a higher risk of STD’s such as HIV [20]. Mass drug administration efforts have thus far not succeeded in eliminating schistosomiasis. This is at least in part due to high re-infection rates after mass drug administration (MDA) with ongoing transmission [21]. A vaccine would greatly contribute to eradication efforts. To this end, a CHIM for *Schistosoma mansoni* (CHI-S) has recently been established at the Leiden University Medical Center in the Netherlands (Fig 1A). Clinical trials have been conducted to firstly set-up the model using male cercariae, secondly, to test the usefulness of female CHI-S and lastly a repeated exposure male CHI-S study investigating the development of protection after repeated infections [16,22,23].

Fresh-water snails of the species *Biomphalaria* are the intermediate host of *S. mansoni*, which are infected by the miracidia that hatch from the eggs deposited in the water through excrements of infected hosts. These snails then shed the cercariae, which are again infective to humans bathing or wading in the water. These cercariae are the infective agents used in this CHIM [24] (Fig 1A).

***Safety:*** Schistosome infections cause pathology in the human host through the deposition of eggs by the mating adult worms. Because this pathology is, in principle, non-reversible, such deposition should be limited as much as possible or prevented altogether. Consequently, the CHI-S model makes use of single-sex cercariae to infect a healthy volunteer. To ensure infection is performed with cercariae of one sex only, a real-time PCR to determine cercarial sex was developed. This PCR targets the *Schistosoma*-specific ITS2 sequence and the *S. mansoni* W1-repeat. Since the W1 repeat is much lower in copy number in male than in female cercariae, the calculated delta between ITS2 cycle-threshold (Ct) value and the W1 Ct value can be used to determine male or female sex of the cercariae [24]. The production process of the infective single-sex cercariae follows the principles of GMP. However, since the production requires a live vector as intermediate host (*Biomphalaria glabrata* snails), it cannot be performed in a full GMP environment.

Adverse events (AE) monitoring is another major aspect which contributes to CHIM safety. In the establishment of novel models in particular, careful dose-escalation is required to detect dose-dependent development of (unexpected) AEs. In the currently performed CHI-S trials, the main adverse events are related to acute schistosomiasis (or Katayama) syndrome and the dermal penetration of cercariae. Thirty-four (34) out of 41 exposed participants in the three currently conducted trials developed a rash at entry site, all characterized as mild [16,22,23]. Symptoms of acute schistosomiasis syndrome seem to be dose-dependent and diminished upon repeated exposures. All three participants exposed to 30 male cercariae developed severe symptoms. With a dose of 20 cercariae, two out of eleven participants in the male model exhibited severe symptoms, which were managed with symptomatic treatment using acetaminophen, non-steroid anti-inflammatory drugs, or prednisone if needed [16]. During steroid treatment (occurring during week 1 or 2 after infection) a temporary decrease in antibody responses was observed, which rebounded after cessation of prednisone. Data on antigen-specific effects of the steroid treatment on cellular immunology to schistosomiasis antigens is not available (Koopman JPR, personal communication). In the female-only experiment, a similar AE profile was observed [22]. In the repeated exposure study, 12 participants were exposed to two repeated infections, treated eight weeks after each infection, and subsequently exposed to third infection to assess protective efficacy. After the first infection, two participants reported severe symptoms of acute schistosomiasis, with another two out of twelve participants in the placebo-immunized group reporting severe symptoms after their first challenge exposure. Notably, symptoms did not increase with repeated exposures but rather seemed to decline in severity [23].

***Detectability:*** The use of a single-sex CHI-S also means that egg detection in feces or urine cannot be used to diagnose infection. Antibodies are another commonly applied diagnostic method, but since antibody titers remain elevated for a prolonged time, even after treatment, they cannot be used to measure treatment effect nor can they quantify the level of infection. Therefore, in CHI-S studies, circulating anodic antigen (CAA) detection using the highly sensitive upconverting phosphor lateral flow (UCP-LF) assay is used as an outcome measure. CAA is an antigen produced in the gut of the schistosome, from which it is regurgitated in the bloodstream of the human host and eventually excreted in the urine. The UCP-LF assay has been developed to pick up extremely low concentrations up to 1 pg/mL of these antigens [25] and can be used on blood or urine. As CAA is produced by the adult worm, it can also be used to track treatment efficacy: after successful treatment antigen levels will be undetectable in blood [26,27]. Using UCP-LF CAA assay, infection could be detected in two out of three participants exposed to 10 male cercariae, in 9 out of 11 exposed to 20 male cercariae and in all three participants exposed to 30 male cercariae [16]. With an apparent attack rate of 82% and an improved safety profile compared to the higher dose, the 20 cercariae model was therefore selected as the preferred protocol for product testing using CHI-S. In the female model, attack rate was found to be similar, with no participants exposed to 10 cercariae reaching detectable CAA levels and 6 out of 10 in the 20 cercariae dose. However, after treatment with praziquantel 60mg/kg at 8 and 12 weeks after infection, 4 out of 10 participants exposed to 20 female cercariae remained CAA positive [22]. In contrast, in the male cercariae model 8 out of 14 participants became undetectable after a first treatment with 40mg/kg praziquantel and all were cleared of infection after a second course of treatment. The repeated infection study, a double blind, randomized, placebo-controlled trial investigated the development of protection against repeated infection, using two infections lasting 8 weeks and subsequently challenging all participants. CAA levels were detectable at 4 weeks after each infection. However, after the third infection, CAA levels were not significantly different in the repeated infection group compared to the placebo group, proving the notion that immunity following repeated infection with schistosomes does not rapidly develop in humans [23].

***Utility:*** Although the CHI-S model was only recently developed, the performed studies have already generated several novel insights into schistosomiasis pathogenesis and immunology. The development of acute schistosomiasis syndrome in the absence of egg development confirmed that, contrary to previously established dogmas, acute schistosomiasis syndrome in humans can occur irrespective of the deposition of eggs [28]. The conducted studies have also generated important insights into the differences between male and female infections in humans, which seem to contradict some of the findings in animal studies. The observation that female cercariae seem to be less susceptible to treatment may be of importance to explain the only limited impact of MDA efforts, as female worms can potentially survive the treatment. The re-exposure study poignantly demonstrates the challenge for schistosome vaccine development: clinical tolerance to infection is rapidly induced in humans, without any sign of protective immunity. However, the rechallenge study also illustrates that the schistosomiasis model is now ready to be applied in vaccine or drug efficacy studies. Currently, the model is only developed for *S. mansoni*. Expanding to other *Schistosoma* species such as S. *haematobium* can have important scientific benefits for vaccine assessment. A single-sex model could, similarly to the *S. mansoni* CHI-S, prevent pathology caused by egg deposition in the urogenital tract by *S. haematobium*, ameliorating safety concerns.

The CHI-S model has further provided insights into the immunological responses after infection. Although no correlates of protection have yet been identified from these studies, this work is ongoing. The studies do show that following an initial Th1-response, the immunological response quickly transition to a Th2-response, as early as eight weeks after infection. This more regulatory response then remains present until infection is cleared [29,30].

Another further development of this model is the transfer to endemic areas, where populations suffer most from these parasitic diseases and would also benefit most from vaccine development. Currently, the CHI-S has only been conducted in Dutch participants, which may result in different immune responses compared to the population in endemic areas. Integration of CHI-S in a vaccine trial would thus boost vaccine development particularly for countries bordering large water bodies such as lake Victoria, Uganda. Conversely, endemic populations add new dimensions to the ethical framework when implementing any CHI model. For the CHI-S model, major considerations include previous and current exposure to *Schistosoma* and co-infections such as malaria that impact host immune responses not only to the CHI-S infection but to a vaccine that would be tested in a CHI-S setting [31]. Particular care is taken to ensure no pre-existent *Schistosoma* infection prior to enrollment in the study; individuals from high-exposure area who test positive at screening will be treated until CAA levels are negative and can only then be reconsidered for inclusion. Individuals from a low-intensity area are excluded from the trial if they test positive at screening. Furthermore, transfer to endemic areas elicits questions regarding spread of non-indigenous strains to the local environment. For *Schistosoma*, this concerns both the parasite and the intermediate snail host which is needed to produce the infective cercariae. Scientists at the Uganda Virus Research Institute have since 2017 taken steps guided by a roadmap to establish a male CHI-S model [32]. The steps being taken include a risk assessment [33] and engagement with the communities with potential volunteers [34]. Also important for endemic settings is to develop critical laboratory and clinical infrastructure and to train local technical human capacity. This has resulted in the development of a specialized snail facility for the parasite culture at the MRC/UVRI and LSHTM Uganda Research Unit in Entebbe. Initial attempts for cercaria production using local Ugandan snails have faced technical challenges and the group has moved to use foreign neotropical snails that have proven successful during the Dutch CHIs (Driciru E. personal communication). This has, however, required a very thorough risk assessment and adaptation of procedures to prevent any risk of foreign snails interfering in the local ecosystems. The establishment of the Uganda CHI-S (ISRCTN14033813) will highlight differences between naïve and exposed populations and a proceeding vaccine-CHI trial will give preliminary efficacy of a selected vaccine candidate in an endemic population.

## Controlled human hookworm infection model (CHHI)

The controlled human hookworm infection model (or CHHI) has a longer history than the CHI-S model, with the first studies performed in the beginning of the 20th century. Historical developments of the hookworm model have previously been reviewed by Chapman and colleagues [10].

Hookworm research highly relies on human challenge models, as the major human hookworm, *Necator americanus*, lacks robust animal models because of its exquisite adaptation to the human host. Infection can be mimicked in hamsters or using the canine hookworm *Ancylostoma caninum* in dogs, however, in all animal models resistance against infection develops that is not reflective of human hookworm infections. This reduces the value of animal models to investigate human hookworm immunology. Although *N. americanus*, *Ancylostoma duodenale*, and *Ancylostoma ceylanicum* cause human pathology, most CHHI models and vaccine development efforts have focused on *Necator*, being the most prevalent species [35]. Infective larvae are cultured from feces of infected human donors that contain the eggs (Fig 1B). These larvae are then applied to the skin of participants (Fig 1B), where they penetrate the skin, migrate through the bloodstream to the lungs, where they are coughed up, swallowed and then end up in the duodenum. There, the larvae mature into the adult worms, where their attachment to the duodenal wall and blood-feeding activities cause anemia and protein losses in vulnerable populations with moderate-to-heavy worm burdens [8]. The preferred challenge doses range from 30 third-stage larvae (L3) to a repeated challenge with 50 L3 twice, depending on study site [10,14,15,36,37].

***Safety:*** In healthy volunteers living in high-income countries, anemia has not been observed, even after prolonged follow-up periods [10,38]. The main symptomatology in CHHI studies is related to either the skin stage of infection or the establishment of the infection in the small intestine, after which infection is usually well tolerated without any residual symptoms.

Severity of skin symptoms is dependent on dose and repeated exposures. Doses of 50 infective larvae (L3) given at one single spot induce more severe skin adverse events [15], whilst spreading the dose over four sites mitigates the effects [38]. Skin symptoms were found to be aggravated after repeated infection, particularly in repeated short-term infections aiming to immunize against larval stages [39]. This skin rash itself has been used as a marker of larval viability in a study using irradiated larvae as an immunization strategy [36]. Abdominal AEs occur when the worms settle in the duodenum, where the larvae mature into adult worms, typically around the fourth week after infection (with a range between 3 and 9 weeks). Symptoms generally persist for 2–4 weeks [10]. In case of severe symptoms, participants can be treated with albendazole, which clears the infection and the symptoms. In dog studies, the lungs were considered an important immunological site [40]. However, significant respiratory symptoms in healthy volunteer studies were not reported. In high doses, mild respiratory symptoms were observed, although there is no documentation of the pulmonary infiltrates and eosinophilia such as can be seen in the classic Loeffler syndrome in *Ascaris* or *Strongyloides* infections [10,41].

***GMP production:*** Hookworm larval culture is currently conducted in five sites worldwide. All sites follow the same production process, although subtle differences in requirements according to national regulations exist. The University of Nottingham was the first site to establish the hookworm culture, using a line of *N. americanus* originally sourced in Papua New Guinea by Prof. David Pritchard of Nottingham University [37]. This lineage has been the starting point for the larval cultures in both Australia at QIMR Berghofer Medical Research Institute and James Cook University, and George Washington University, Washington DC, United States. Both sites use procedures similar to the original culture in Nottingham, although all sites slightly adapt to meet local requirements. In Australia, larvae are produced according to GMP principles; however, according to the national regulatory agency, products used in Phase I testing do not need to comply with full GMP, challenge agents are furthermore not considered ‘therapeutics’ and exempted from full GMP manufacturing [42]. A similar procedure is used in Wellington, New Zealand [43,44], where also GMP methods for cryopreservation of hookworm larvae are developed. In the United States, the infective larvae are considered a biological product and must therefore comply with the applicable legislation. Here, the procedure has been adapted to meet the requirements for studies under an investigational new drug (IND) application [15,45]. In the Netherlands, larvae are cultured in a similar manner as in Australia and were originally derived from an Australian donor. In the European Union, challenge agents are considered Auxiliary Medicinal Products for which full GMP production is not required although preferred [38].

For the transfer of the CHHI model to Lambaréne in Gabon, a highly endemic area, a novel challenge strain has been cultured. Due to concerns about the introduction of a non-native strain into the environment, the initial culture was started using eggs from feces of a locally infected person [46]. This prevents the risk of spread of a different strain into the local environment. The development of this novel strain again highlights the questions that need to be considered upon transfer to endemic areas.

***Detectability:*** To detect vaccine efficacy, a reliable measure of infection is needed. Egg excretion in feces is the most frequently used endpoint for CHHI studies. This can be done through quantitative microscopy, using the gold standard Kato-Katz method, or through more sensitive PCR detection or using hatching assays where larvae are cultured from the feces [10]. Although reliable for detecting viable infections, all methods suffer from high variability in their quantification, reducing study power of any vaccine or drug study. As sterile immunity against infection is not considered a feasible goal for a hookworm vaccine, assessment of vaccine efficacy will rely on effectuating a significant decrease in worm burden after vaccination. Reducing egg output variability is therefore important to increase the study power. Modeling egg excretion over a prolonged period provides a better assessment of infection burden and consequently can improve study power [14].

Hookworm infection intensity in CHHI trials varies, but doses between 50 L3 and 150 L3 reach levels comparable to a mild-moderate natural infection [14,39]. Next to the use of Kato-Katz or other microscopic techniques, infection can be measured using PCR detection or through coproculture using hatching assays. PCR testing has been found to correlate well with microscopy and was found to be a more sensitive diagnostic tool in microscopy-negative individuals [36]. Nevertheless, the PCR test also suffers from day-to-day variability in egg density. Detection of antibodies to recombinant excretory/secretory proteins has been described [47,48], but these findings have not culminated in the development of a clinically validated serodiagnostic test.

Hatching assays can assess the viability of eggs but also rely heavily on egg excretion in the feces, consequently this measure is equally variable. Some studies have used capsule endoscopy to visualize adult worms attached to the duodenal wall, however, as these interventions are costly and may not provide visualization of all worms present, detection of hookworm infection intensity remains an estimate rather than an exact count.

***Utility:*** The CHHI model has been used for both possible anti-inflammatory effects in inflammatory and metabolic disorders and as a tool for vaccine efficacy testing. Trials studying anti-inflammatory effects have shown mixed results, although most showed at least some effect on disease outcomes. Persons with metabolic syndrome showed decreased insulin resistance after hookworm infection [13], and trials in patients with celiac disease showed improved symptom scores and effector-regulatory T cell ratios, but inconsistent pathology at biopsies [12,49]. In asthma patients, however, only a non-significant decrease in airway hyperresponsiveness was observed [11], noting that this study utilized very low doses of challenge L3. These results may partially be explained by the significantly lower doses administered (10–40 L3) than in immunization trials and in experimental compared to natural infections, or may be due to inherent disease characteristics that have not yet been elucidated. As described above, several studies have resulted in improvements of the model to be better suited for efficacy testing. The model has since been applied in two different immunization studies. A study using irradiated larvae as the immunogen and a study using a repeated infection and treatment approach, both followed by challenge, resulted in the first proof of any protective efficacy in humans against hookworm challenge [36,39]. As both these studies expose participants to larval stages only, this has now revived interest in the larval stage of infection as a possible source for antigens that can be applied in a subunit vaccine. Samples from these studies form a highly valuable treasure trove that is now to be explored using antigen arrays [6,50]. These studies have also provided more information on immunological responses against repeated infection. Although still exploratory in nature, both studies have shown increases in IgG1 responses in vaccinated individuals, related to decreased egg production in the repeated abrogated infection study [39]. Furthermore, a mixed cytokine response was observed after vaccination and challenge with increases in both Th1 and Th2 cytokines [36]. A study of immunological responses following long-term infection in hookworm donors showed development of a regulatory T-cell response, although not as pronounced as compared to natural infection [51].

Following the development of allergic reactions to the hookworm aspartic protease (*Na-*ASP-2) vaccine in pre-exposed individuals [52], any larval stage antigen would necessitate extensive screening in pre-exposed individuals to ensure that existing IgE responses (as a result of prior or current infection) are not contributing to vaccine-induced hypersensitivity responses. Furthermore, efforts are underway to use the CHHI model to test the efficacy of the only hookworm vaccine currently in clinical development, the aspartic protease 1(APR1)/gluthathone S-transferase-1(GST1) vaccine, results of which are pending (NCT03172975).

The hookworm model was successfully transferred to Gabon, a highly endemic area. Similarly to the CHI-S, implementation started with a thorough risk analysis and extensive engagement activities [46]. Successful establishment of the culture at the CERMEL research facilities has resulted in the execution of the first hookworm infection trial in an endemic area. Results of the first infections in this population are currently pending (PACTR202202553002020). This model will provide further insights into immune responses in pre-exposed populations and can provide highly important information on vaccine efficacy in the target population.

## CHI-S and CHHI: differences and similarities

**Fig 2:**
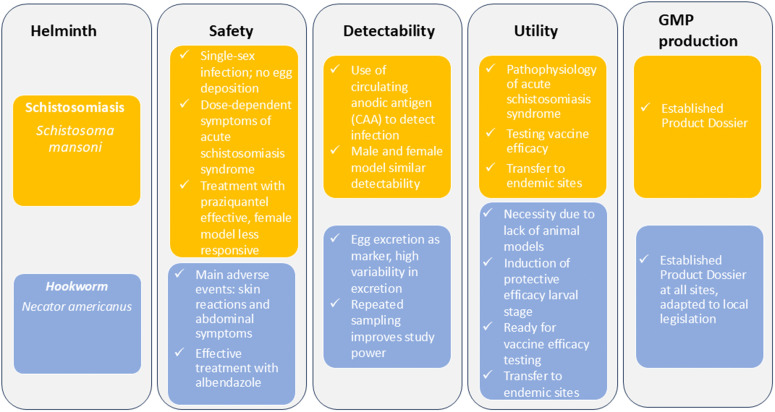
Key points for the development and use of the controlled human schistosoma and hookworm infection models.

Fig 2 provides a brief summary of the key elements of the discussed helminth-controlled human infection models. Both the hookworm and the schistosome-controlled human infection models are generally well-tolerated, but do cause contrasting symptomatology. In the hookworm model, the symptoms are initially skin-related and then abdominal symptoms can occur following establishment of the adult worms in the gut. These gastro-intestinal symptoms can be managed symptomatically with pain killers or anti-emetics, but, if unsuccessful, infection needs to be cleared with albendazole. In the schistosomiasis model, skin symptoms are less pronounced, although still frequently present. Most symptoms in the schistosome infection studies relate to acute schistosomiasis syndromes. These inflammatory symptoms can be dampened with anti-inflammatory drugs such as non-steroidal anti-inflammatory drugs and prednisone and do not necessitate abrogation of the infection. After the acute infection phase, both models cause asymptomatic infection in the long term. Both helminths have well-proven treatments to clear infection, although the success rate of albendazole treatment for hookworm is nearly 100% in the CHHI studies described, whereas praziquantel treatment for schistosomiasis may need to be repeated and is, particularly in the female model, not 100% effective.

Both models have limitations in their comparability to natural infection. This partly relates to the previously discussed differences in what population is typically included in the CHIMs versus the target population of the vaccines. This is partly overcome by transfer to endemic areas, where the role of co-infections on immunological responses can also be better assessed. Furthermore, natural infections are usually characterized by repeated exposure to low numbers of infective larvae as compared to single-dose administrations in CHIM.

Infection levels in the controlled hookworm model can reach those of mild to moderate endemic areas in CHHI studies which use higher infectious doses. In the schistosome infections, CAA levels are manifold lower than those seen in endemic areas due to the dose-dependent development of acute schistosomiasis syndrome. In addition, unlike natural infection, due to the single-sex nature of the schistosome infections, eggs cannot be detected.

A marked difference between the hookworm and schistosomiasis CHI models is the clear contrast between the immunological responses upon repeated infection. Whereas in the hookworm infection, skin reactions increased in severity with subsequent exposures and protective effects can be seen, this was not the case in repeated schistosome infections where clinical tolerance rapidly occurred [23,39]. These differing responses highlight the heterogeneity of helminth infections, particularly when comparing intravascular schistosomes with intraduodenal hookworms. Alternatively, immune responses may also be dictated in part by treatment schedules: the hookworm infection was abrogated before hookworms reached the adult stage, whereas in the schistosome model treatment was initiated when adult forms were already present. Particularly, adult worms are known for their immune modulatory capacity. On the other hand, schistosomes induced a much more intense, dose-dependent initial immune response whereby a much lower inoculum of cercariae can already induce inflammatory syndromes, whereas even high larval doses are generally well-tolerated in hookworm infection.

## What are the remaining questions in hookworm and schistosomiasis CHIM development?

Several important questions remain to be investigated. First, current trials have been undertaken in a non-exposed population. Given the difference in immune responses in endemic populations compared to non-exposed individuals, the transfer of these models to endemic sites in Gabon and Uganda will be insightful for vaccine development [8].

Another unaddressed question is the role of co-infections. Helminth infections often occur in concurrence with other infections, which may impact immunological responses [31]. However, whether such responses may be synergistic or mitigating helminth immune manipulation remains elusive. Combining the hookworm and schistosomiasis CHIM models provides exciting possibilities to investigate this, although a careful evaluation of potential burden for participants would be needed to assure a positive burden-benefit balance.

Although both models are now well-established and suited for vaccine- and drug efficacy trials, the first results of trials with compounds in clinical development are still awaited or have not even started yet. Repeated immunization studies have shown that both the hookworm and schistosomiasis model are useful and adapted to investigating vaccine- or drug efficacy testing.

## Concluding remarks

Both the controlled infection model for hookworm as well as schistosomes can be considered safe, leading to adequately detectable infections with multiple use-cases, with GMP-like production being established for both models. For both models, specific methodological challenges have been overcome, related to the detectability, treatment, and burden for participants. Both models have some limitations in their comparability to natural infections, particularly related to the challenge inoculum dose and timing and for the schistosome model the lack of egg production. The planned establishment of a *Trichuris* controlled infection model is another stepping stone into furthering the possibilities for helminth vaccine development, which, using CHIMs, has made important steps forward for schistosomiasis and hookworm. As the value and safety of these helminth CHIMs have been clearly established, this may pave the way for further development of helminthic CHIMs.

**Key learning points**

- Both the controlled human schistosomiasis and hookworm infection models have proven safe, detectable, and have shown usefulness in the vaccine development pathway
- These two different helminth CHI have demonstrated marked differences in human host immune responses to short-term infection, highlighting the challenges in developing effective vaccines.
- Remaining questions include transfer of the models to endemic areas, further research into co-infections, and further expansion of the portfolio helminthic challenge models.

**Key papers**

- Koopman JPR, Houlder EL, Janse JJ, Lamers OA, Roozen GV, Sijtsma JC, et al. Clinical tolerance but no protective efficacy in a placebo-controlled trial of repeated controlled schistosome infection. The Journal of clinical investigation. 2024;135(4 *First demonstration of the use of the CHI-S model as model to test efficacy of immunisation strategy.*
- Chapman PR, Webster R, Giacomin P, Llewellyn S, Becker L, Pearson MS, et al. Vaccination of human participants with attenuated *Necator americanus* hookworm larvae and human challenge in Australia: a dose-finding study and randomised, placebo-controlled, phase 1 trial. The Lancet Infectious diseases. 2021;21(12):1725–36 *First demonstration of protective efficacy against hookworm infection after exposure to irradiated larvae in humans.*
- Abaasa A, Egesa M, Driciru E, Koopman JPR, Kiyemba R, Sanya RE, Nassuuna J, Ssali A, Kimbugwe G, Wajja A, van Dam GJ, Corstjens PLAM, Cose S, Seeley J, Kamuya D, Webb EL, Yazdanbakhsh M, Kaleebu P, Siddiqui AA, Kabatereine N, Tukahebwa E, Roestenberg M, Elliott AM. Establishing a single-sex controlled human *Schistosoma mansoni* infection model for Uganda: protocol for safety and dose-finding trial. Immunother Adv. 2023 Jul 20;3(1):ltad010 *Protocol paper for the development of the CoHSI model in Uganda.*
- Alabi A, Hussain M, Hoogerwerf MA, Mengome CN, Egesa M, Driciru E, et al. Establishing a controlled hookworm human infection (CHHI) model for Africa: A report from the stakeholders meeting held in Lambaréné, Gabon, November 10–11, 2019. Archives of public health Archives belges de sante publique. 2021;79(1):120 R*eport on the development of the CHHI-model in Gabon.*
- Erwin G, Scholte L, Saes R, Li G, Schellhaas L, Ratnappan R, Pritchard DI, Hawdon J, Diemert D, Bethony JM. Manufacture of *Necator americanus* as an infectious challenge agent: Accelerating human hookworm vaccine development. Microb Pathog. 2025 Jul;204:107592. *Paper extensively describing the GMP production process of hookworm larvae.*
